# Relationship between the ratio of erythrocyte distribution width to albumin level and mortality in hypertensive population: Mediating role of inflammatory markers

**DOI:** 10.1371/journal.pone.0324027

**Published:** 2025-05-23

**Authors:** Dongli Huang, Chun Zou, Hang Wu

**Affiliations:** Bishan Hospital of Chongqing Medical University, Chongqing, China; Duke University Medical Center: Duke University Hospital, UNITED STATES OF AMERICA

## Abstract

**Background:**

This study examined the ratio of erythrocyte distribution width (RDW) to albumin concentration (RAR) and all-cause, cardiovascular disease (CVD), and cancer mortality in the hypertension population, focusing on the role of inflammatory markers as mediators.

**Patients and methods:**

Data from NHANES (1999–2018) were analyzed, linking National Death Index (NDI) records to mortality outcomes through December 31, 2019. A weighted sampling design categorized participants into three RAR groups. Cox regression models adjusted for demographic and clinical variables assessed the association between RAR and mortality outcomes. Mediation analyses explored the mediating role of the systemic Inflammatory response index (SIRI) and neutrophil-to-lymphocyte ratio (NLR).

**Results:**

Among 26,935 participants with a median follow-up of 102 months and 6,007 deaths, elevated RAR was associated with increased risks of all-cause mortality (HR = 1.83, 95% CI: 1.76–1.90), cardiovascular disease mortality (HR = 1.81, 95% CI: 1.68–1.95), and cancer mortality (HR = 1.70, 95% CI: 1.55–1.86). Segmented regression showed a nonlinear relationship between RAR and all-cause mortality, cardiovascular mortality, and cancer mortality, and the threshold effect results showed a fold value of 4.10, with a greater HR when RAR < 4.10. Mediation analysis revealed that SIRI and NLR mediated the relationship between RAR and all-cause mortality by 8.12% and 6.00%, respectively.

**Conclusion:**

In hypertensive populations, higher RAR values are associated with increased all-cause mortality, cardiovascular mortality, and cancer mortality. Inflammation partially mediates the relationship between RAR and all-cause mortality.

## Introduction

Hypertension is a global epidemic, currently affecting approximately 1.13 billion people [1]. Individuals with hypertension are at a significantly higher risk of death compared to the general population, with hypertension being a leading cause of death worldwide, accounting for approximately 9.4 million deaths annually [2]. Thus, identifying prognostic indicators that can pinpoint high-risk individuals is crucial for improving clinical outcomes in hypertensive patients [3].

In recent years, erythrocyte distribution width (RDW) and albumin (ALB) have gained significant clinical attention as important biomarkers. RDW reflects erythrocyte volume variability, serving as a sensitive indicator closely related to the onset and prognosis of various chronic diseases, such as cardiovascular disease, diabetes mellitus, and chronic kidney disease [4–6]. Albumin, the major plasma protein, plays a critical role in maintaining colloid osmotic pressure and reflects the body’s nutritional status. Low albumin levels are commonly associated with chronic inflammation, malnutrition, and disease progression, which contribute to a poor prognosis. As a composite biomarker, RAR effectively reflects various physiological states, including chronic inflammation, oxidative stress, and malnutrition. Hao et al. found that high RAR was strongly associated with mortality in the general population [7]. Other studies have explored its association with mortality in specific populations, particularly those with chronic inflammatory conditions such as rheumatoid arthritis, diabetes mellitus, and diabetic retinopathy [5,8,9]. However, the association between RAR and prognosis in hypertensive patients remains underexplored. Therefore, this study aimed to investigate the relationship between RAR and prognosis in hypertensive patients and explore the role of inflammation in this process.

## Methods

### Data and study participants

**Fig 1:**
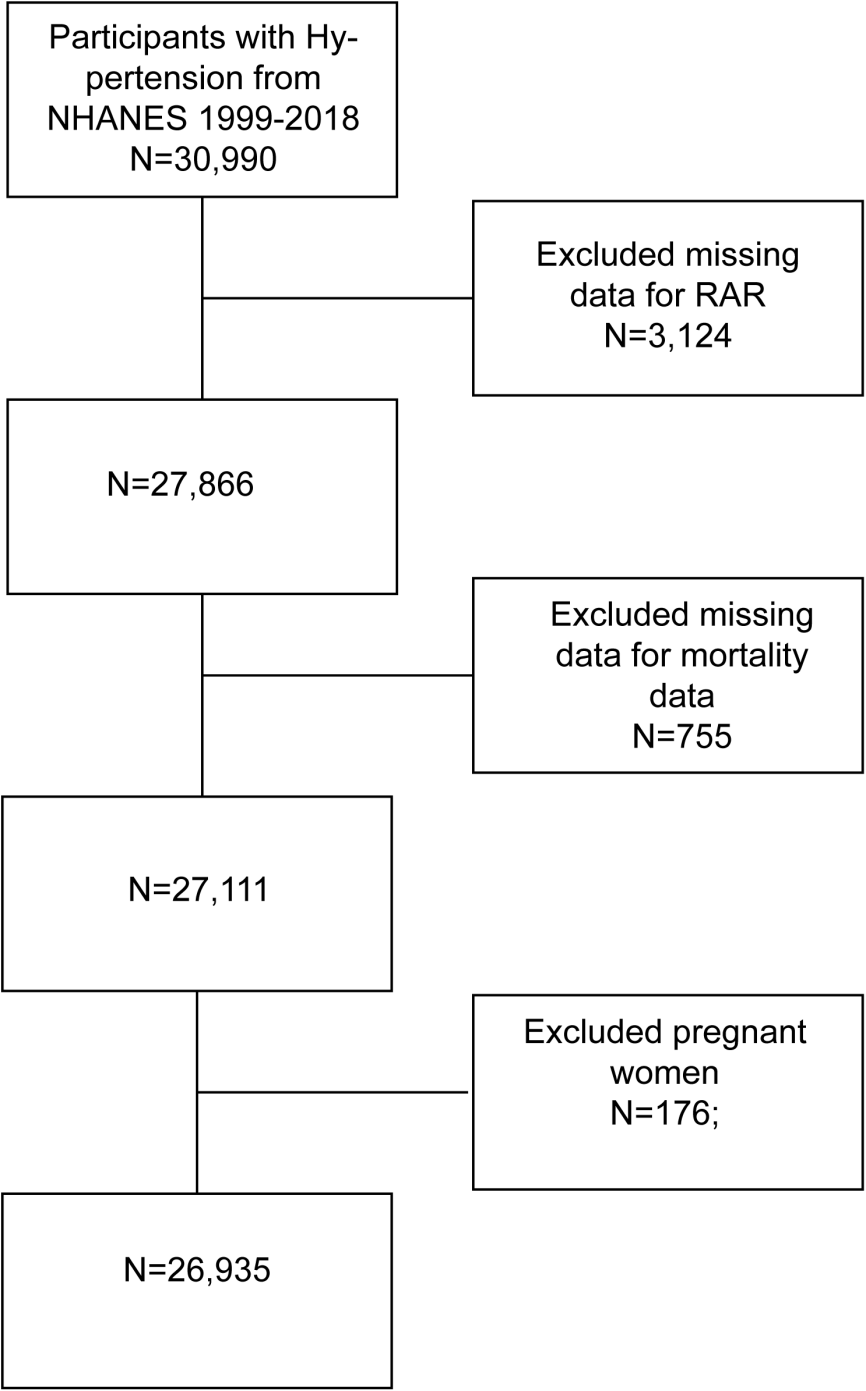
Flowchart of the study population.

The National Health and Nutrition Examination Survey (NHANES) is a population-based study designed to collect health and nutritional data from a representative sample of the U.S. household population. Data were obtained through a multistage probability sampling approach, encompassing structured household interviews, physical examinations conducted at mobile examination centers, and laboratory assessments. This study utilized de-identified data from NHANES, a publicly accessible database managed by the National Center for Health Statistics (NCHS), a division of the Centers for Disease Control and Prevention (CDC). The NHANES data collection protocol was approved by the NCHS Research Ethics Review Board (ERB). Given that this study involved secondary analysis of anonymized, publicly available data, it was exempt from additional Institutional Review Board (IRB) review. Informed consent was obtained from all NHANES participants by the NCHS during the original data collection, and no further consent was required for this analysis. A total of 30,990 hypertensive patients were identified from the NHANES 1999–2018 dataset. Specifically, based on NHANES database information, hypertension was defined as systolic blood pressure ≥130 mmHg, diastolic blood pressure ≥80 mmHg, a physician diagnosis of high blood pressure, or current use of prescription medication for hypertension. We excluded 3,124 individuals missing RDW or albumin (Alb) values required for the RAR formula. Additionally, 755 respondents were excluded due to missing final survival status data. To ensure result reliability, 176 pregnant women were excluded. The complete data screening process is illustrated in Fig 1. All data used in this study were publicly available (https://www.cdc.gov/nchs/nhanes/) and were weighted based on demographic characteristics for subsequent analysis.

### Definition of the ratio of erythrocyte distribution width to albumin index

In the NHANES study, serum albumin concentration was measured using the bromocresol purple method.RDW was measured using a Coulter analyzer in mobile examination centers with peripheral blood samples. RAR was calculated using the formula RDW/Alb, as described in previous studies [10].

### Definition of the the systemic inflammatory response index and the neutrophil–lymphocyte ratio

Neutrophil, lymphocyte, and monocyte counts were measured using automated hematology analyzers.The systemic Inflammatory response index (SIRI) is calculated as: SIRI = (Monocyte count × Neutrophil count)/ Lymphocyte count. The neutrophil–lymphocyte ratio (NLR) is calculated as: NLR = Neutrophil count/ Lymphocyte count.

### Outcome ascertainment

All-cause mortality was assessed using National Death Index (NDI) records linked to the NHANES dataset, with follow-up data available through December 31, 2019. Cause-specific mortality was classified based on ICD-10 codes. Cardiovascular disease (CVD) mortality was defined by ICD-10 codes I00–I09, I11, I13, and I20–I51. Cancer mortality was defined by ICD-10 codes C00–C97.

### Assessment of covariates

The covariates in this study included age(<45,45–60, >60), gender, race, education level, marital status, household poverty-to-income ratio (PIR), body mass index (BMI), physical activity (vigorous or moderate), smoking status, alcohol consumption, diabetes mellitus, hypercholesterolemia, and cardiovascular disease (CVD). Physical activity was categorized as vigorous (yes/no) or moderate (yes/no). Participants who reported smoking at least 100 cigarettes in their lifetime and continued smoking at the time of the survey were defined as current smokers. Former smokers were defined as individuals who had smoked at least 100 cigarettes in their lifetime but had quit smoking by the time of the survey. Additionally, individuals who had smoked fewer than 100 cigarettes in their lifetime were classified as nonsmokers. Nondrinkers were participants who reported not consuming at least 12 alcoholic beverages in their lifetime or any given year. In this study, individuals who reported consuming 12 alcoholic beverages in their lifetime or within any year but abstained from alcohol in the past 12 months were classified as former drinkers. Participants who reported consuming 12 alcoholic beverages in their lifetime or any year and drank at least one alcoholic beverage in the past 12 months were defined as current drinkers. Participants were considered diabetic if they had been diagnosed by a physician, had a fasting blood glucose level ≥7.0 mmol/L, glycosylated hemoglobin ≥6.5%, a 75 g oral glucose tolerance test result ≥200 mg/dL, or were using glucose-lowering medications or insulin. Hypercholesterolemia was defined as total cholesterol >5.2 mmol/L, low-density lipoprotein cholesterol (LDL-C) >3.4 mmol/L, or the use of lipid-lowering medications. CVD was defined as a prior diagnosis of congestive heart failure, coronary artery disease, angina, stroke, or myocardial infarction.

### Statistical analysis

All statistical analyses in this study accounted for the multi-stage NHANES design by incorporating appropriate sampling weights, strata, and primary sampling units. In the baseline characteristics table, continuous variables were presented as weighted means with standard errors (SE), and categorical variables as weighted proportions. The Kruskal-Wallis rank sum test was applied to continuous variables, and the survey-weighted chi-square test was used for categorical variables, to evaluate differences across RAR tertiles. Multivariate Cox regression was employed to examine the relationship between RAR and all-cause, cardiovascular disease, and cancer mortality. Three models were employed: Model 1 (unadjusted); Model 2 (adjusted for age, sex, and ethnicity); and Model 3 (adjusted for age, sex, ethnicity, body mass index, education, marital status, PIR, hypercholesterolemia, diabetes mellitus, alcohol consumption, physical activity, cardiovascular disease, and smoking). We applied smoothed curve fitting and generalized additive modeling (GAM) to examine the nonlinear association between RAR and all-cause and cause-specific mortality. When nonlinear correlations were detected, we fitted a two-stage linear regression model (segmented regression) to each interval to assess threshold effects. The breakpoint (K) was identified using a two-step recursive method (S1 File). Mediation analyses were conducted to explore the potential mediating roles of two inflammatory markers (SIRI and NLR) in the relationship between RAR and all-cause mortality, adjusting for factors in the primary analysis Model 3.All statistical analyses were conducted using EmpowerStats (http://www.empowerstats.com, X&Y Solutions, Inc.) and R statistical software (http://www.R-project.org; The R Foundation).

## Results

### Association between RAR and mortality

As shown in Table 1, the study included 26,935 participants aged 18 years or older, with 52.72% being male. The all-cause mortality rate was 22.30% over a median follow-up of 102 months. Baseline characteristics for each RAR stratum are presented in Table 1. Participants with higher RAR levels were more likely to be over 60 years old and to have diabetes or cardiovascular disease.

**Table 1:** Baseline characteristics of NHANES participants from 1999-2018. Classified according to RAR tertiles.

Variables	Tertiles of RAR			*P value*
	T1	T2	T3	
N	8949	8991	8995	
Follow-up time,(months)	138.80 ± 63.28	108.26 ± 61.39	82.08 ± 58.22	<0.001
Age	51.00 ± 17.47	58.57 ± 16.21	60.55 ± 15.70	<0.001
SII	540.74 ± 313.52	537.34 ± 314.38	614.82 ± 563.56	<0.001
SIRI	1.22 ± 0.80	1.27 ± 0.86	1.45 ± 1.19	<0.001
NLR	2.15 ± 1.09	2.18 ± 1.13	2.41 ± 1.57	<0.001
PLR	128.65 ± 48.69	126.80 ± 50.38	135.00 ± 65.57	<0.001
PPN	1079.10 ± 551.38	1071.73 ± 578.94	1176.72 ± 1142.80	<0.001
RAR	2.75 ± 0.14	3.10 ± 0.10	3.72 ± 0.51	<0.001
LDL, (mmol/L)	3.14 ± 0.92	3.02 ± 0.95	2.85 ± 0.95	<0.001
HDL, (mmol/L)	1.34 ± 0.42	1.35 ± 0.42	1.35 ± 0.43	0.006
TC, (mmol/L)	5.30 ± 1.10	5.16 ± 1.10	4.91 ± 1.13	<0.001
TG, (mmol/L)	1.75 ± 1.33	1.65 ± 1.23	1.48 ± 1.30	<0.001
All-cause mortality				<0.001
No	7418 (82.89%)	6948 (77.28%)	6562 (72.95%)	
Yes	1531 (17.11%)	2043 (22.72%)	2433 (27.05%)	
Cardiovascular mortality				<0.001
No	8567 (95.73%)	8464 (94.14%)	8288 (92.14%)	
Yes	382 (4.27%)	527 (5.86%)	707 (7.86%)	
Cancer mortality				<0.001
No	8603 (96.13%)	8531 (94.88%)	8555 (95.11%)	
Yes	346 (3.87%)	460 (5.12%)	440 (4.89%)	
Gender, n(%)				<0.001
Male	5761 (64.38%)	4688 (52.14%)	3750 (41.69%)	
Female	3188 (35.62%)	4303 (47.86%)	5245 (58.31%)	
Age,n(%)				<0.001
<45	3294 (36.81%)	1853 (20.61%)	1574 (17.50%)	
>=45, < 60	2418 (27.02%)	2325 (25.86%)	2167 (24.09%)	
>=60	3237 (36.17%)	4813 (53.53%)	5254 (58.41%)	
PIR				<0.001
<1.3	2213 (24.73%)	2414 (26.85%)	2947 (32.76%)	
>=1.3, < 3.5	3796 (42.42%)	4050 (45.05%)	4138 (46.00%)	
>=3.5	2940 (32.85%)	2527 (28.11%)	1910 (21.23%)	
BMI, n(%)				<0.001
<25	2487 (27.79%)	1899 (21.12%)	1573 (17.49%)	
>=25, < 30	3648 (40.76%)	3240 (36.04%)	2612 (29.04%)	
>=30	2814 (31.44%)	3852 (42.84%)	4810 (53.47%)	
Race, n(%)				<0.001
Mexican American	1696 (18.95%)	1394 (15.50%)	1099 (12.22%)	
Other Hispanic	600 (6.70%)	765 (8.51%)	663 (7.37%)	
Non-Hispanic White	4691 (52.42%)	4190 (46.60%)	3333 (37.05%)	
Non-Hispanic Black	1185 (13.24%)	1896 (21.09%)	3206 (35.64%)	
Other Race	777 (8.68%)	746 (8.30%)	694 (7.72%)	
Education, n(%)				<0.001
Under high school	2330 (26.04%)	2609 (29.02%)	2789 (31.01%)	
High school or equivalent	2047 (22.87%)	2207 (24.55%)	2175 (24.18%)	
College graduate or above	4572 (51.09%)	4175 (46.44%)	4031 (44.81%)	
Marital Status, n(%)				<0.001
Married or living with partner	5877 (65.67%)	5550 (61.73%)	4706 (52.32%)	
living alone	3072 (34.33%)	3441 (38.27%)	4289 (47.68%)	
Smoke, n(%)				0.029
Current smokers	1638 (18.30%)	1672 (18.60%)	1794 (19.94%)	
Nonsmokers	4722 (52.77%)	4665 (51.89%)	4579 (50.91%)	
Former smokers	2589 (28.93%)	2654 (29.52%)	2622 (29.15%)	
Diabetes, n(%)				<0.001
No	7520 (84.03%)	6810 (75.74%)	5877 (65.34%)	
Yes	1429 (15.97%)	2181 (24.26%)	3118 (34.66%)	
Vigorous activity, n(%)				<0.001
No	5722 (63.94%)	6597 (73.37%)	7182 (79.84%)	
Yes	3227 (36.06%)	2394 (26.63%)	1813 (20.16%)	
Moderate activity, (n%)				<0.001
No	4929 (55.08%)	5466 (60.79%)	6072 (67.50%)	
Yes	4020 (44.92%)	3525 (39.21%)	2923 (32.50%)	
Drink, n(%)				<0.001
Current drinkers	6484 (72.46%)	5739 (63.83%)	5209 (57.91%)	
Nondrinkers	975 (10.90%)	1290 (14.35%)	1393 (15.49%)	
Former drinkers	1490 (16.65%)	1962 (21.82%)	2393 (26.60%)	
CVD				<0.001
No	8071 (90.19%)	7513 (83.56%)	6829 (75.92%)	
Yes	878 (9.81%)	1478 (16.44%)	2166 (24.08%)	
Hypercholesterolemia, n(%)				<0.001
No	2829 (31.61%)	3223 (35.85%)	3823 (42.50%)	
Yes	6120 (68.39%)	5768 (64.15%)	5172 (57.50%)	

RAR Erythrocyte distribution width to albumin ratio, PIR poverty-to-income ratio, BMI body mass index, SII systemic immune-inflammation index, TC total cholesterol, TG triglyceride, LDL-C low-density lipoprotein cholesterol, HDL-C high-density lipoprotein cholesterol.

### Association between RAR and mortality

As shown in Table 2, higher RAR levels were significantly associated with an increased risk of all-cause mortality in Model 1 (HR = 2.08, 95% CI: 2.01–2.15). The association remained robust and statistically significant after multivariate adjustments in Model 2 (HR = 2.06, 95% CI: 1.99–2.13) and Model 3 (HR = 1.83, 95% CI: 1.76–1.90). Participants were categorized into three groups according to RAR tertiles. Compared with the first tertile, participants in the third tertile exhibited a 119% increase in all-cause mortality, a 125% increase in cardiovascular mortality, and a 78% increase in cancer mortality. The trend across tertiles was statistically significant (trend p < 0.0001).

**Table 2:** Association of RAR with mortality in the population.

	Model 1	Model 2	Model 3
	HR 95% CI	HR 95% CI	HR 95% CI
**All-cause mortality**	2.08 (2.01, 2.15)	2.06 (1.99, 2.13)	1.83 (1.76, 1.90)
T1	Ref	Ref	Ref
T2	1.83 (1.71, 1.96)	1.44 (1.35, 1.54)	1.34 (1.25, 1.44)
T3	3.07 (2.88, 3.28)	2.63 (2.46, 2.82)	2.19 (2.04, 2.35)
*P* for trend	<0.0001	<0.0001	<0.0001
**Cardiovascular mortality**	2.12 (2.00, 2.25)	2.08 (1.96, 2.22)	1.81 (1.68, 1.95)
T1	Ref	Ref	Ref
T2	1.89 (1.66, 2.16)	1.47 (1.29, 1.68)	1.33 (1.16, 1.52)
T3	3.57 (3.14, 4.05)	2.99 (2.62, 3.42)	2.25 (1.96, 2.58)
*P* for trend	<0.0001	<0.0001	<0.0001
**Cancer Mortality**	1.89 (1.74, 2.04)	1.85 (1.70, 2.02)	1.70 (1.55, 1.86)
T1	Ref	Ref	Ref
T2	1.78 (1.55, 2.05)	1.43 (1.24, 1.65)	1.36 (1.18, 1.57)
T3	2.37 (2.05, 2.73)	2.02 (1.74, 2.35)	1.78 (1.53, 2.08)
*P* for trend	<0.0001	<0.0001	<0.0001

HR: hazard ratio.

95% CI: 95% confidence interval.

Model 1: no covariates were adjusted.

Model 2: Adjusted for age, gender, and race.

Model 3: Age, gender, race, body mass index, education, marital status, PIR, diabetes, alcohol consumption, vigorous activity, moderate activity, cardiovascular disease, smoking, hyper cholesterol.

**Fig 2:**
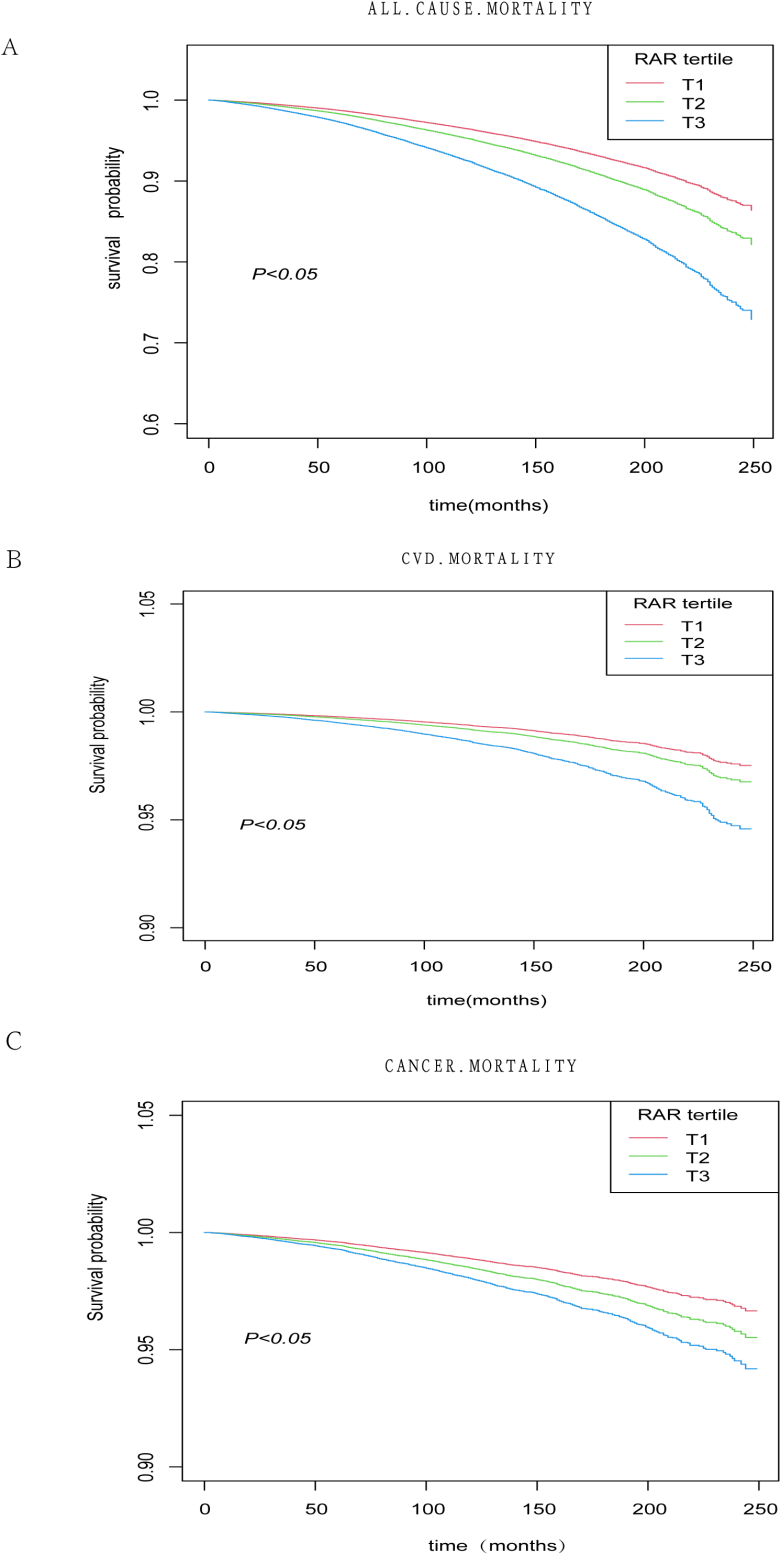
Kaplan-Meier survival curves for the effect of RAR on long-term all-cause(A) and cardiovascular disease(B), and cancer(C) mortality in the hypertension population.

**Fig 3:**
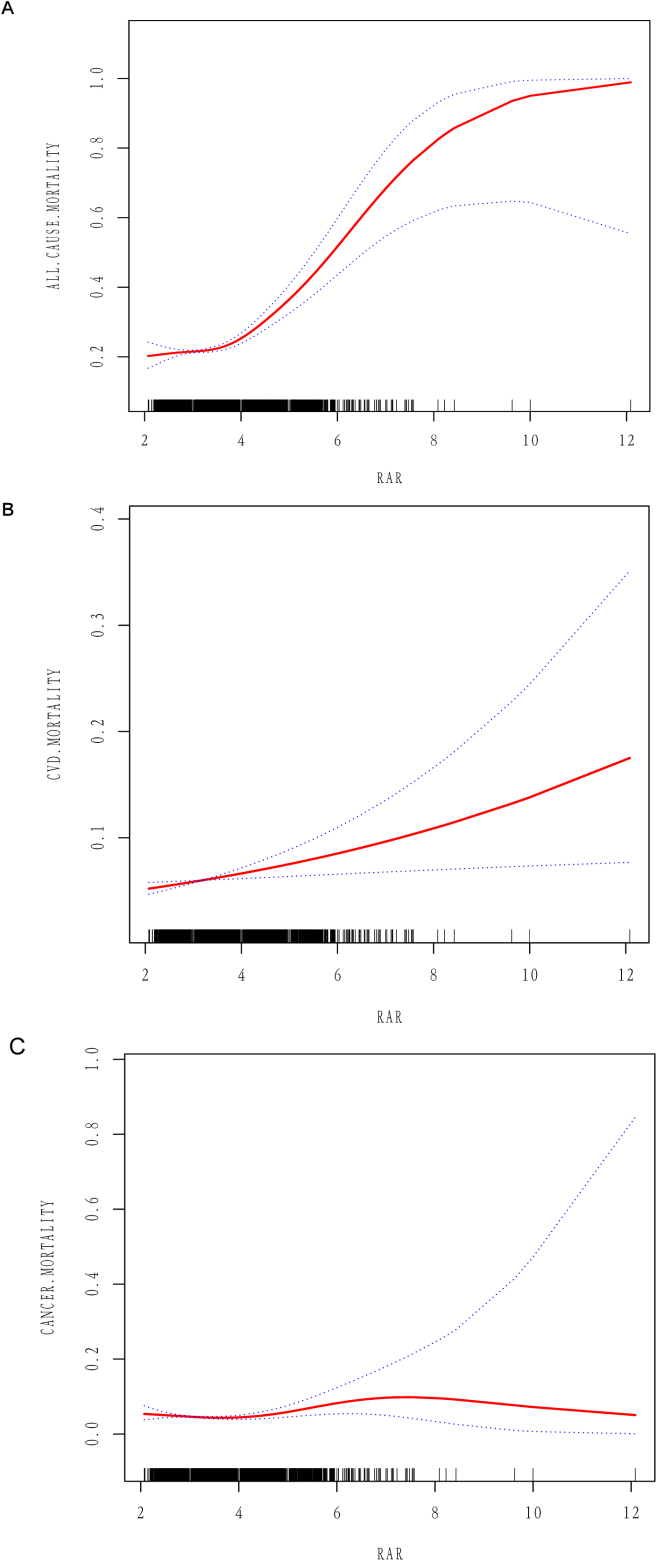
Association between RAR and hypertension population-based all-cause mortality (A), cardiovascular disease (B), and cancer (C) mortality. Adjustments have been made for age, sex, race, body mass index, education, marital status, PIR, hypercholesterolemia, diabetes mellitus, alcohol consumption, vigorous activity, moderate activity, cardiovascular disease, and smoking.

The Kaplan-Meier curve (Fig 2) supports these findings. The curve illustrates that in the hypertensive population, participants with higher RAR levels experienced a greater decline in survival during follow-up compared with those with lower RAR levels (log-rank p < 0.05). Fig 3 demonstrates that after full adjustment for confounders and smooth curve fitting, a positive association was observed between RAR and all-cause, cardiovascular, and cancer mortality. Threshold effects analysis showed that HR was greater when the RAR was less than 4.1 (Table 3).

**Table 3:** Threshold effects of RAR on mortality analyzed using linear regression models.

	Adjusted HR (95% CI), P Value
**RAR vs All-cause mortality**	
Fitting by the standard linear model	1.82(1.76, 1.89) <0.0001
Fitting by the two-piecewise linear model	
RAR	
Inflection point	4.10
RAR < 4.10	2.47(2.30, 2.65) <0.0001
RAR >4.10 Log likelihood ratio	1.35(1.24, 1.47) <0.0001 <0.001
**RAR vs CVD mortality**	
Fitting by the standard linear model	1.81(1.68, 1.95) <0.0001
Fitting by the two-piecewise linear model	
RAR	
Inflection point	4.10
RAR < 4.10	2.44(2.12, 2.79) <0.0001
RAR >4.10 Log likelihood ratio	1.31(1.10, 1.56) 0.0029 <0.001
**RAR vs cancer mortality**	
Fitting by the standard linear model	1.69(1.54, 1.85) <0.0001
Fitting by the two-piecewise linear model	
RAR	
Inflection point	4.10
RAR < 4.10	1.97(1.68, 2.32) <0.0001
RAR >4.10	1.42(1.18, 1.72) 0.0003
Log likelihood ratio	0.0019

### Sensitivity analysis

We incorporated age as a continuous covariate in the Cox regression analyses, which revealed (S1 Table) no substantial change in study outcomes, with HR values showing consistent effect directions and variations within 3%. Age-stratified analyses revealed that, among hypertensive patients, RAR and mortality exhibited consistent associations with statistically significant P-values across all age groups (<45, 45–60, > 60 years) (S2 Table).

### The mediating role of SII and SIRI

**Fig 4:**
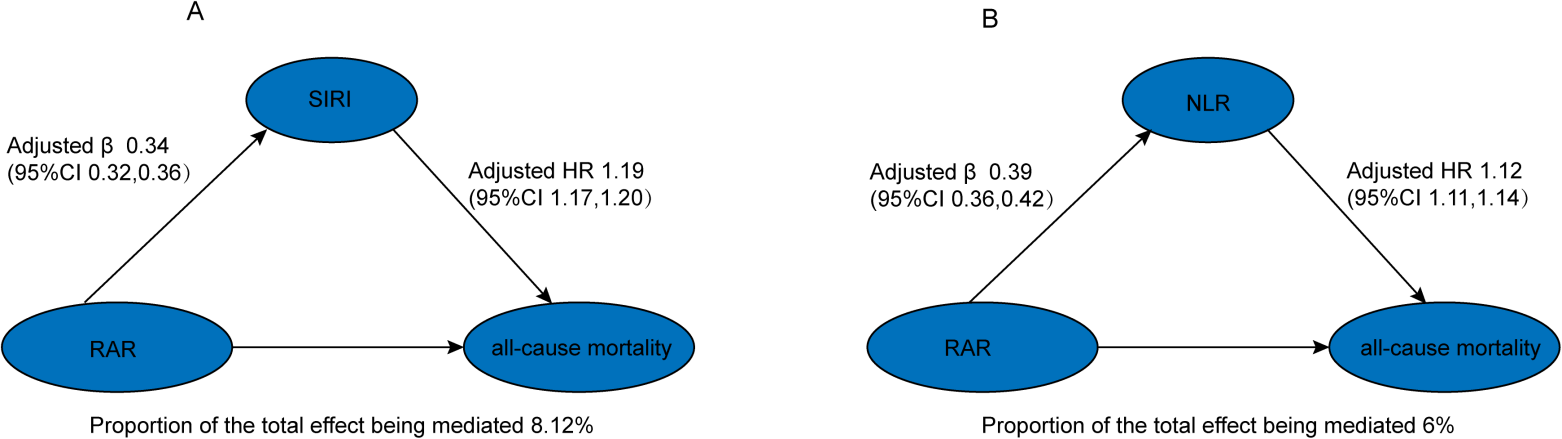
Role of SIRI and NLR in the association between RAR and all-cause mortality. (A) Mediation analysis of the association between RAR and all-cause mortality by SlRl. (B) Mediation analysis of the association between RAR and all-cause mortality by NLR.

High levels of RAR are associated with increased all-cause mortality. To investigate the mediating role of inflammation in the relationship between RAR and all-cause mortality among a hypertensive population, we conducted mediation analyses using SIRI and NLR as markers of systemic inflammatory status. In the fully adjusted model, RAR was correlated with SIRI (β = 0.34, 95% CI: 0.32–0.36). Likewise, in the fully adjusted model, RAR was positively correlated with NLR (β = 0.39, 95% CI: 0.36–0.42). Subsequently, multivariate Cox regression analyses were performed to examine the associations between SIRI, NLR, and all-cause mortality. Results indicated that in the fully adjusted model, SIRI was positively associated with all-cause mortality (HR = 1.19, 95% CI: 1.17–1.20), as was NLR (HR = 1.12, 95% CI: 1.11–1.14). Finally, mediation effect analyses (Fig 4 and Table 4) revealed that, after adjusting for all covariates, the relationship between RAR and all-cause mortality was mediated by SIRI and NLR by approximately 8.12% and 6.00%, respectively. Additionally, other inflammatory markers, including the aggregate index of systemic inflammation (AISI), platelet-to-lymphocyte ratio (PLR), and systemic immune-inflammation index (SII), exhibited partial mediation when analyzed for mediating effects (S3 Table).

**Table 4:** The mediating role of SIRI and NLR in the association between RAR and all-cause mortality.

Mediation effect	Estimate	95% CI lower	95% CI upper	*P*-value
**SIRI**				
Total effect	131.43	119.49	144.43	<0.0001
Mediation effect	10.73	9.07	12.57	<0.0001
Direct effect	122.57	110.76	135.11	<0.0001
Proportion mediated	0.08	0.06	0.09	<0.0001
**NLR**				
Total effect	136.92	124.72	150.23	<0.0001
Mediation effect	8.27	6.97	9.67	<0.0001
Direct effect	128.65	116.59	141.31	<0.0001
Proportion mediated	0.06	0.05	0.07	<0.0001

Age, gender, race, body mass index, education, marital status, PIR, hypercholesterolemia, diabetes, alcohol consumption, vigorous activity, moderate activity, cardiovascular disease, smoking were adjusted.

## Discussion

This study investigated the association between the red cell distribution width to albumin ratio (RAR) and mortality in hypertensive individuals, with a particular focus on the mediating role of inflammatory markers. Our findings revealed that higher RAR values were significantly associated with increased mortality. Notably, the strength of this association was more pronounced when RAR values were below 4.1.

These findings align with prior studies demonstrating the prognostic significance of RDW and albumin levels in chronic diseases [7–9]. RDW, a marker of red cell heterogeneity, is associated with oxidative stress and systemic inflammation, while hypoalbuminemia reflects malnutrition and an ongoing inflammatory state. By integrating these two markers, RAR provides a more comprehensive reflection of the inflammatory and nutritional status of individuals.

Inflammation appears to be a pivotal mechanism linking RAR to mortality in hypertensive individuals. Chronic low-grade inflammation is a well-established contributor to the pathophysiology of cardiovascular and metabolic diseases, as it drives processes such as endothelial dysfunction, vascular remodeling, and atherosclerosis [11–14]. In our study, the SIRI and NLRmediated 8.12% and 6.00% of the association between RAR and all-cause mortality, respectively. These findings underscore the partial but significant role of systemic inflammation in amplifying mortality risk among hypertensive patients [11,15].

Inflammatory markers such as C-reactive protein (CRP) and interleukin-6 (IL-6) are frequently elevated in hypertensive individuals and play critical roles in this context [16,17]. CRP is an acute-phase protein that reflects systemic inflammation and directly contributes to endothelial dysfunction by inhibiting nitric oxide production, increasing oxidative stress, and promoting vascular stiffness [18,19]. Similarly, IL-6 acts as both a pro-inflammatory cytokine and a regulator of acute-phase responses, exacerbating vascular inflammation and contributing to arterial plaque instability. These processes may lead to vasoconstriction, increased thrombogenesis, and elevated blood pressure, creating a self-perpetuating cycle of inflammation and vascular injury [12,15,20,21].

Moreover, inflammation can impair metabolic homeostasis by influencing lipid metabolism and reducing insulin sensitivity. Elevated inflammatory cytokines have been shown to increase lipolysis, promote the formation of oxidized low-density lipoproteins (ox-LDL), and impair glucose uptake in peripheral tissues. For instance, the presence of inflammatory cytokines such as interleukin-6 and tumor necrosis factor-alpha has been positively correlated with metabolic disturbances, including insulin resistance and dyslipidemia, which are critical factors in the pathogenesis of cardiovascular diseases [22]. Additionally, macrophages, which play a crucial role in atherosclerosis, can ingest ox-LDL, leading to foam cell formation—a hallmark of atherosclerotic plaques [23,24]. This process is exacerbated by the inflammatory environment, which further promotes lipid metabolism disorders and insulin resistance [25].

These metabolic derangements not only worsen the cardiovascular risk profile of hypertensive patients but may also directly contribute to the observed association between the erythrocyte distribution width-to-albumin ratio (RAR) and mortality. RAR has been identified as a potential prognostic marker in various cardiovascular conditions, including heart failure and diabetic complications [9,26]. Studies have shown that elevated RAR levels correlate with increased mortality rates, suggesting that inflammation and metabolic dysregulation may mediate this relationship [27,28]. Furthermore, the inflammatory milieu associated with atherosclerosis can lead to increased production of pro-inflammatory cytokines and reactive oxygen species, which in turn can impair glucose metabolism and exacerbate cardiovascular risks [29,30]. Thus, the interplay between inflammation, lipid metabolism, and insulin sensitivity is critical in understanding the mechanisms linking RAR to mortality in hypertensive patients.

The observed threshold effect indicates a nonlinear association between RAR and mortality, with a higher hazard ratio (HR) when RAR is below 4.10 compared to when it exceeds 4.10. In hypertensive patients with RAR > 4.10 (higher RAR group), this may indicate that inflammatory trophic homeostasis has entered a decompensation phase, where predominant end-stage events (e.g., sudden cardiac death, multi-organ failure) could obscure RAR’s independent effect amid a worsened physiological state. Nevertheless, additional mechanistic studies are required to elucidate this association. Monitoring RAR levels could aid in stratifying patients based on their mortality risk and guiding targeted interventions. Reducing RAR values may mitigate inflammation, improving vascular health and overall prognosis.

Despite its strengths, this study has several limitations. First, the retrospective design may have introduced selection bias, limiting causal inferences. Although we adjusted for multiple potential confounders, residual confounding from unmeasured factors, such as socioeconomic status or specific treatment regimens, cannot be excluded. Second, while NHANES data are representative of the U.S. population, their applicability to other regions or ethnic groups may be limited.

Future research should confirm these findings across diverse populations, including hypertensive patients from various regions and with differing comorbidities, to assess RAR’s generalizability as a prognostic biomarker. Longitudinal and repeated-measures studies with extended follow-up are also required to clarify the causal links between RAR, inflammation, and mortality. The threshold effect at RAR = 4.10 necessitates additional molecular biology studies to uncover the underlying mechanisms. Lastly, clinical trials assessing the efficacy of anti-inflammatory interventions in patients with elevated RAR levels could yield valuable insights into strategies for improving prognosis in this high-risk group.

## Conclusions

In hypertensive populations, elevated RAR values are associated with increased all-cause, cardiovascular, and cancer-related mortality. Systemic inflammation partially mediates the relationship between RAR and all-cause mortality, highlighting its role as a potential link between nutritional status, inflammation, and adverse outcomes.

## Supporting information

S1 Raw data Original data for this study. (CSV)

S1 File Threshold and saturation effect analysis explained. (DOCX)

S1 Table Association of RAR with mortality in the population. (DOCX)

S2 Table Subgroup Analysis of the Relationship Between RAR and Mortality by Age. (DOCX)

S3 Table Mediating role of other inflammatory markers in the association between RAR and all-cause mortality. (DOCX)
