# Supplementary material for: Relationship between the ratio of erythrocyte distribution width to albumin level and mortality in hypertensive population: Mediating role of inflammatory markers
Source: PLoS One. 2025 May 23;20(5):e0324027. doi: 10.1371/journal.pone.0324027 (PMC12101704; doi:10.1371/journal.pone.0324027)
Supplement: S1 File — (DOCX) [file pone.0324027.s002.docx]

**S1 File: Threshold and saturation effect analysis explained**

First, we applied smooth curve fitting to examine whether the independent variable, RAR, could be partitioned into intervals. Then, we used segmented regression (also known as piecewise regression), which fits separate line segments to each interval. To determine if a threshold exists, we performed a log-likelihood ratio test, comparing the non-segmented model (single line) to the segmented regression model.

Next, we identified the inflection point (the point where the segments connect) by maximizing the model’s likelihood using a two-step recursive method:

Step 1:

We narrowed down the potential inflection point to a 10th percentile range of the independent variable. Specifically, we tested 19 segmented regression models using percentile points from 5% to 95%, incremented by 5%. For each of the 19 models, we tested different percentile points as potential inflection points and selected the one with the highest likelihood. We then refined the range of the inflection point to within +/- 4% of the percentile point that yielded the highest likelihood, which we referred to as Kmin and Kmax.

Step 2:

To determine the precise inflection point, we performed a recursive method within the narrowed range of Kmin and Kmax. In this step, we ran three models using inflection points at the 25% (Q1), 50% (Q2), and 75% (Q3) percentiles of the narrowed range. We selected the quartile point that provided the highest likelihood and further reduced the range of Kmin and Kmax to +/- 25% of that quartile point. This recursive process continued until we identified the precise inflection point that gave the highest likelihood for the segmented regression model.
