# Supplementary material for: Relationship between the ratio of erythrocyte distribution width to albumin level and mortality in hypertensive population: Mediating role of inflammatory markers
Source: PLoS One. 2025 May 23;20(5):e0324027. doi: 10.1371/journal.pone.0324027 (PMC12101704; doi:10.1371/journal.pone.0324027)
Supplement: S1 Table — (DOCX) [file pone.0324027.s003.docx]

S1 Table: Association of RAR with mortality in the population

|  | **Model 1**  **HR 95% CI** | **Model 2**  **HR 95% CI** | **Model 3**  **HR 95% CI** |
| --- | --- | --- | --- |
| **All-cause mortality** | 2.08 (2.01, 2.15) | 2.00 (1.92, 2.07) | 1.78 (1.71, 1.85) |
| **Cardiovascular mortality** | 2.12 (2.00, 2.25) | 2.02 (1.88, 2.16) | 1.76 (1.62, 1.91) |
| **Cancer**  **Mortality** | 1.89 (1.74, 2.04) | 1.80 (1.64, 1.97) | 1.65 (1.49, 1.81) |

HR: hazard ratio

95% CI: 95% confidence interval

Model 1: no covariates were adjusted

Model 2: Adjusted for age, gender, and race

Model 3: Age (continuous variable), gender, race, body mass index, education, marital status, PIR, diabetes, alcohol consumption, vigorous activity, moderate activity, cardiovascular disease, smoking, hypercholesterol.
