# Supplementary material for: Relationship between the ratio of erythrocyte distribution width to albumin level and mortality in hypertensive population: Mediating role of inflammatory markers
Source: PLoS One. 2025 May 23;20(5):e0324027. doi: 10.1371/journal.pone.0324027 (PMC12101704; doi:10.1371/journal.pone.0324027)
Supplement: S2 Table — (DOCX) [file pone.0324027.s004.docx]

S2 Table: Subgroup Analysis of the Relationship Between RAR and Mortality by Age

| **Character** | **HR 95% CI** |  | ***P*-value** |
| --- | --- | --- | --- |
| **All-cause mortality**  Age |  |  |  |
| <45  45-60 | 2.30 (1.86 2.83)  2.30 (2.12 2.50) |  | <0.001  <0.001 |
| >60 | 1.77 (1.70 1.84) |  | <0.001 |
| **Cardiovascular mortality** |  |  |  |
| Age  <45  45-60  >60  **Cancer mortality** | 2.84 (1.96 3.55)  2.27 (1.90 2.72)  1.80 (1.66 1.94) |  | <0.001  <0.001  <0.001 |
| Age |  |  |  |
| <45 | 1.60 (0.98, 2.61) |  | 0.0582 |
| 45-60 | 1.72 (1.36, 2.19) |  | <0.001 |
| >60 | 1.71 (1.56, 1.89) |  | <0.001 |
