# Supplementary material for: Relationship between the ratio of erythrocyte distribution width to albumin level and mortality in hypertensive population: Mediating role of inflammatory markers
Source: PLoS One. 2025 May 23;20(5):e0324027. doi: 10.1371/journal.pone.0324027 (PMC12101704; doi:10.1371/journal.pone.0324027)
Supplement: S3 Table — (DOCX) [file pone.0324027.s005.docx]

S3 Table: Mediating role of other inflammatory markers in the association between RAR and all-cause mortality.

| **Mediation effect** | **Estimate** | **95% CI lower** | **95% CI upper** | ***P*-value** |
| --- | --- | --- | --- | --- |
| **AISI** |  |  |  |  |
| Total effect | 137.50 | 125.45 | 150.90 | <0.0001 |
| Mediation effect | 6.45 | 5.35 | 7.63 | <0.0001 |
| Direct effect | 132.25 | 120.23 | 145.33 | <0.0001 |
| Proportion mediated | 0.047 | 0.039 | 0.056 | <0.0001 |
| **PLR** |  |  |  |  |
| Total effect | 142.41 | 129.75 | 155.82 | <0.0001 |
| Mediation effect | 5.56 | 4.04 | 7.23 | <0.0001 |
| Direct effect | 137.92 | 124.88 | 151.23 | <0.0001 |
| Proportion mediated  **SII**  Total effect  Mediation effect  Direct effect  Proportion mediated | 0.039  139.50  5.94  134.70  0.042 | 0.028  127.36  4.80  122.44  0.035 | 0.051  152.88  7.17  147.79  0.052 | <0.0001  <0.0001  <0.0001  <0.0001 |

Age, gender, race, body mass index, education, marital status, PIR, hypercholesterolemia, diabetes, alcohol consumption, vigorous activity, moderate activity, cardiovascular disease, and smoking were adjusted.
